# Editorial: Tactile Intelligence in Robots

**DOI:** 10.3389/fnbot.2020.00056

**Published:** 2020-09-18

**Authors:** Sung-Phil Kim, Christian Wallraven, Vincent Duchaine

**Affiliations:** ^1^Department of Biomedical Engineering, Ulsan National Institute of Science and Technology, Ulsan, South Korea; ^2^Department of Artificial Intelligence, Department of Brain and Cognitive Engineering, Korea University, Seoul, South Korea; ^3^École de Technologie Supérieure, Montreal, QC, Canada

**Keywords:** robot object manipulation, tactile sensation and perception, tactile learning, dexterous skill, somatosensory system

Tactile intelligence has become increasingly important in the development of intelligent robots capable of dexterity skills that are comparable to those in humans. Recent advances in tactile sensors have fostered the implementation of artificial tactile sensation in robots, where tactile intelligence plays a key role in translating physical signals from tactile sensors to tactile percepts. Amid many possible approaches to the development of tactile intelligence, it is reasonable to translate the principles of information processing of the human nervous system into artificial robot intelligence. For instance, one may create artificial neural networks that mimic somatosensory nervous systems and integrate them with advanced machine learning techniques in order to have robots gain human-like dexterity skills. This Research Topic aims to highlight state-of-the-art research on the implementation of tactile intelligence in robots inspired by neural mechanisms of tactile information processing. It also emphasizes human studies on tactile perception to provide insight on robot learning for object manipulation tasks.

A number of studies contributed to the present Research Topic by providing the latest remarkable findings in tactile intelligence. One of the technical challenges in the implementation of tactile intelligence in robots is ensuring the robots can sense external mechanical stimulations with high fidelity. Unlike the widely distributed mechanoreceptors inside the human hand, the current tactile sensing technology in robotic hands often suffers from a sparseness of sensing points and a lack of spatial resolution. Sun and Martius attempt to overcome this limitation by inferring tactile stimulations at virtual contacts from a limited number of strain-gauged sensor data using machine learning algorithms. They demonstrate the feasibility of reconstructing the location and force magnitudes of deformable objects at multiple contact points from sparse sensory configurations. They achieve this by leveraging machine learning algorithms for the inference of tactile information, clearly showing how robots gain the ability of inference from limited observations, akin to what human intelligence does.

Another study by Richardson and Kuchenbecker contributing to this topic investigates more closely such connections between robot tactile sensing and human tactile perceptual attributes. They particularly focus on attribute intensity and perceptual variability in natural human tactile perception, which is not available in the current robot tactile intelligence. They collected haptic adjectives for a number of objects from human subjects as well as robotic tactile sensing data for the same objects. Then they successfully predicted the probability distribution of haptic adjectives from the tactile sensing data of an object using a machine-learning algorithm. The study demonstrates the possibility of modeling both the intensity and variability of human tactile perception using tactile intelligence in robots. This finding will enable artificial tactile intelligence to move closer to the human perceptual system.

Tactile intelligence will become increasingly important as robots begin to function in more open-ended environments, where they will have to adapt to the uncertainty of environmental states. In this regard, Seminara et al. reviewed sensorimotor coupling in the robotic control of hands and fingers with an emphasis on connections between human tactile perception and robotic tactile sensing. In particular, they highlight robotic behavior, goals, and tasks in active haptic exploration. This review offers a comprehensive review of the taxonomy of elements for the closed-loop sensorimotor control of robots and will be of great help to those who seek to design a robot with the ability to interact with various real environments adaptively and intelligently.

Closing the loop of motor control with sensory feedback can lead to the cognitive embodiment of external actuators in humans. This is especially important to the use of robots as an assistive technology in a daily life. Beckerle et al. provides a critical review on the role of tactile perception of sensory feedback in the feeling of embodiment while using assistive robotic systems. Throughout the rigorous review, they suggest practical solutions to enhance embodiment by optimizing tactile feedback in human-robot interactions. This review will be of considerable value to those who aim to improve the usability of robotic assistive technology by providing real-time tactile feedback to the user.

The first review by Seminara et al. discusses the role of tactile intelligence in a closed sensorimotor loop for robotic control of hands and fingers, and the second review by Beckerle et al. deals with how such a sensorimotor loop could induce the feelings of embodiment when using robot-based assistive technology. So, the understanding of a sensorimotor loop using tactile feedback in the first review can provide a basis for the realization of embodiment in assistive technology in the second review. Based on these reviews, we believe that the upcoming issue in the field will be developing robotic upper limbs and tactile sensors that are equipped with the sophisticated model of the closed sensorimotor loop and practical applications of these advanced technologies to real-life situations, such as assistive environments.

We believe that all the contributions to this topic will broaden our understanding of the neural underpinnings of tactile perception, foster the development of robots interacting with the world in a more intelligent way, and open a new avenue to integrate multiple disciplines in order to pave the way for next-generation human-robot interactions. We hope that readers will also find in this Research Topic useful insights and promising outlooks for their own research.

## Author Contributions

S-PK edited and reviewed the contributions, and wrote the Editorial. CW and VD edited and reviewed the contributions. All authors contributed to the article and approved the submitted version.

## Conflict of Interest

The authors declare that the research was conducted in the absence of any commercial or financial relationships that could be construed as a potential conflict of interest.

